# Gut microbiota are differentially correlated with blood pressure status in African American collegiate athletes: A pilot study

**DOI:** 10.14814/phy2.15982

**Published:** 2024-03-21

**Authors:** Taylor Hogue, Jarrad Hampton‐Marcell, Ian M. Carroll, Troy Purdom, Heather Colleran, T. J. Exford, Michael Brown, Marc D. Cook

**Affiliations:** ^1^ Department of Kinesiology North Carolina Agriculture and Technical State University Greensboro North Carolina USA; ^2^ Department of Biological Sciences University of Illinois at Chicago Chicago Illinois USA; ^3^ Department of Nutrition University of North Carolina at Chapel Hill Chapel Hill North Carolina USA; ^4^ Department of Nutrition North Carolina Agriculture and Technical State University Greensboro North Carolina USA; ^5^ Education & Research Department Dayton VA Medical Center Dayton Ohio USA; ^6^ Department of Kinesiology University of Maryland College Park Maryland USA; ^7^ Center for Integrative Health Disparity & Equity Research (CIHDER) North Carolina Agricultural and Technical State University Greensboro North Carolina USA

**Keywords:** African American, athlete, gut microbiome, hypertension

## Abstract

Hypertension (HTN) is common among athletes and the most recent epidemiologic data reports that cardiovascular (CV) sudden death is significantly greater in African Americans (AAs). Gut microbial dysbiosis (a poorly diverse stool microbial profile) has been associated with HTN in sedentary people but microbial characteristics of athletes with HTN are unknown. Our purpose was to differentiate microbiome characteristics associated with BP status in AA collegiate athletes. Thirty AA collegiate athletes were stratified by normal BP (systolic BP (SBP) ≤130 mmHg; *n* = 15) and HTN (SBP ≥130 mmHg; *n* = 15). 16S rRNA gene sequencing was performed on stool samples to identify microbes at the genus level. We did not observe any significant differences in alpha diversity, but beta diversity was different between groups. Principal coordinate analysis was significantly different (PERMANOVA, *p* < 0.05, *R* = 0.235) between groups. Spearman rank correlations showed a significant (*p* < 0.05) correlation between systolic BP and abundances for *Adlercreutzia* (*R* = 0.64), *Coprococcus* (*R* = 0.49), *Granulicatella* (*R* = 0.63), and *Veillonella* (*R* = 0.41). Gut microbial characteristics were associated with differentially abundant microbial genus' and BP status. These results will direct future studies to define the functions of these microbes associated with BP in athletes.

## 1 INTRODUCTION

Hypertension (HTN) is a global health concern and the risk of one developing HTN in the United States is approximately 90% (Vasan et al., 2002) affecting all ethnic groups. It is commonly understood that African Americans (AAs) have a higher risk of developing HTN (Benjamin et al., 2019). Interestingly, HTN is not uncommon among athletes (Leddy & Izzo, 2009) and there has been discussion regarding the most appropriate pharmacological treatment strategies to reduce this risk in athletes and preserve performance (Oliveira & Lawless, 2010). The most recent demographic and epidemiologic data shows that elevated blood pressure (BP) in athletes is associated with an increased risk of cardiovascular (CV) sudden death (Maron et al., 2016). In the most recent review, this phenomenon was reported to be 5‐times more common in AA athletes compared to whites (1:60 vs. 1:12,778, 746 athlete‐years) (Maron et al., 2016). Reasons for this disparity are unclear but studies, such as this, are needed to define potential factors. Overall, cardiometabolic fitness (e.g., VO_2_max) is associated with lower BP (Sui et al., 2017). Although chronic exercise (i.e., aerobic and anaerobic) and greater cardiometabolic fitness is associated with a reduction in BP (Boutcher & Boutcher, 2017; Pal et al., 2013), athletes with HTN have an elevated risk of experiencing cardiac dysfunction precipitating sudden CV death and this makes HTN among athletes' a significant health concern.

Interest in the functionality of the gut microbiota in human health and disease, including cardiovascular disease (CVD) has increased (Belizario & Faintuch, 2018; Tang et al., 2017). CVDs, such as HTN, are associated with multiple factors that include but are not limited to obesity, smoking, age, and family history (Tsao et al., 2023). The human gut microbiome is composed of diverse populations of microbes (Ahmad et al., 2019) and is also impacted by lifestyle (i.e., diet (Hills Jr. et al., 2019) and exercise (Mailing et al., 2019)), and other environmental factors (Cresci & Bawden, 2015). The community of bacteria are important in dictating metabolic processes that involve processing food while synthesizing various nutrients and metabolic byproducts that facilitate their absorption into the systemic circulation and passage through the bowel (Fukui et al., 2018). HTN is associated with gut microbial dysbiosis, which is consistently characterized by the stool microbiome expressing decreased diversity (lower alpha and beta diversity) (Kriss et al., 2018) and altered short‐chain fatty acid (SCFA) production capacity among various racial/ethnic populations (de la Cuesta‐Zuluaga et al., 2018; Yang et al., 2015). SCFAs (i.e., butyrate, propionate, and acetate) are products of gut microbial fermentation of dietary fibers in the colon and are absorbed into the blood, via transporter proteins (Sivaprakasam et al., 2017). In the United States, gut dysbiosis associated with HTN consists of blunted SCFA production in sedentary populations (Yang et al., 2015) and maladaptive metabolism of foods that contribute to the persistence of inflammatory responses in CVD (Li et al., 2017). Participation in regular exercise is inversely related to CVD risk and can reduce BP (Pescatello et al., 2019; Saco‐Ledo et al., 2020). Our previous work, and the work of others, have shown that regular exercise can also promote increased gut microbial diversity and SCFA production in animals (Cook et al., 2016) and humans (Allen et al., 2018; Mailing et al., 2019). In relation to HTN, exercise likely has synergistic effects in the vasculature and gut microbiome that translates to improved vascular health outcomes (Cook & Hogue, 2021).

Research characterizing the microbiome of athletes shows optimal microbial diversity associated with greater efficiency in amino acid and carbohydrate metabolism, and enhanced SCFA production (Barton et al., 2018) which was associated with fitness, overall health, and more recently with athletic performance (Clauss et al., 2021; Furber et al., 2022). To date, there has been limited published research on microbial profiles associated with HTN in athletes. This underrepresentation means data quantifying gut microbial characteristics and specifying microbial relationships with BP status in athletes is crucial to provide insight on what gut dysbiosis is in this special population.

In this study, our objective was to assess stool microbial characteristics (e.g., microbial diversity, abundances of microbes associated with BP status, and functional metabolic pathways) in AA athletes, with and without HTN. To date, there are too few research studies targeting AA athletes, who are disproportionately impacted by multiple CVD risk factors. From the existent studies on sedentary gut dysbiosis and HTN, we hypothesized that AA athletes with HTN might also exhibit lower microbial diversity and lower abundance of SCFA producing microbes.

## 2 MATERIALS AND METHODS

### 2.1 Subjects

Thirty AA collegiate athletes from North Carolina A&T State University teams (men's basketball (11 participants), track and field (11 participants), women's volleyball (six participants), and Men's football (two participants)) completed participation in this study. The participants were a mix of aerobic and power/strength trained athletes. As this was a pilot study, we were unable to conduct a power analysis for this study as there is no data available related to the novel relationship(s) between gut microbiome characteristics and HTN in athletes. This study was approved by the North Carolina A&T State University Institutional Review Board (IRB: 17–148). All participants completed written informed consent before data collection. Inclusion criteria for this study included male and female subjects between 18 and 25 years of age. Study participants were members of the athletic program and participated in training and competition for their respective sport in the previous year. All athletes were tested at the start of their preseason summer training programs and were not competitively active within the previous 14 days prior to testing. Participants indicated that they were not taking any prescription medications, nor had they taken over‐the‐counter pre‐workout stimulants within the last 14 days or probiotic supplements or antibiotics within 3 months of participating in the study. BP and body composition data were collected from subjects in the morning before any exercise training activity (no exercise for at least 12 h) and with at least a 2‐hour fasting period (no food or drink). A 3‐day diet recall was collected 3 days prior to the testing day. Results of the diet recall are recently published here (Purdom et al., 2023). Stool samples were collected on the 3rd day of the diet recall within 24 h of the testing day.

### 2.2 Blood pressure

On the testing day, participants BP were recorded manually after a 5‐minute resting period in the supine position. BP (measured between 8 am and 11 am) was taken twice, 1 min apart, and the average BP is reported. If the initial systolic or diastolic BP readings had a difference of 5 mmHg or more apart, a 3rd measurement was recorded, and the last two readings were averaged. BP was taken by the same individual for all participants to reduce variability. Participants were categorized as having normal BP (systolic BP ≤129 mmHg) or hypertension (systolic BP ≥130 mmHg) per the updated American Heart Association guidelines (Benjamin et al., 2019).

### 2.3 Body composition measurement

Height was measured using a stadiometer (Seca; Chino, CA) and body composition was measured using bioelectrical impedance (Seca mBCA 514; Chino, CA). Body weight, body mass index (BMI), fat mass, fat‐free mass, and total body water are reported (Table 1). All subjects refrained from exercise (at least 12 hours), food and fluid intake for at least 2 h before the bioelectrical impedance measurement.

**TABLE 1:** Describes the characteristics of athletes with normal blood pressure and hypertension.

	Normotensive *n* = 15, 8F	Hypertensive *n* = 15, 4F	*p*‐Value
Age (Years)	19.53 ± 1.24	19.53 ± 1.48	0.692
Weight (lbs.)	153.54 ± 23.84	195.73 ± 48.1	0.0051**
Height (in.)	68.7 ± 4.43	72.6 ± 4.63	0.0248*
Body Mass Index status	14 N, 1 O	9 N, 3 O, 3 OB	N/A
Fat Mass (%)	17.02 ± 7.07	20.86 ± 10.15	0.2415
Muscle Mass (lbs.)	63.22 ± 13.39	77.74 ± 15.24	0.0098**
Systolic (mmHg)	119.8 ± 3.68	134.73 ± 10.83	<0.0001***
Diastolic (mmHg)	75.3 ± 4.28	81.6 ± 6.47	0.0086**
VO_2_ (L/min)	3.76 0.85	4.79 1.28	0.014*
VO_2_ (mL/kg/min)	53.84 ± 6.85	56.04 ± 16.33	0.637

*Note*: Data are reported as mean ± SD.

Abbreviations: N, Normal BMI; OB, obese; O, overweight.

*p < 0.05; **p < 0.01; ***p < 0.001.

### 2.4 Maximal oxygen consumption (VO_2_max)

VO_2_max was measured (after the BP and body composition assessment) with a modified Bruce protocol graded treadmill exercise test (Parvo Medics True Max 2400 metabolic cart). Subjects heart rate (HR) was taken continuously using a wireless Polar Monitor H10 (Polar Electro, Lake Success, NY) and subjects continued until they met at least three of four criteria for the maximum aerobic capacity test (HR ± 10 beats of predicted HRmax; respiratory exchange ratio ≥ 1.15; plateau in oxygen consumption despite an increase in intensity; volitional fatigue). Relative VO_2_max (milliliters (mL) of O_2_ per kilogram (kg) of body weight per minute; mL/kg/min) is reported.

### 2.5 Gut microbiome

Subjects submitted swabs of stool samples to assess microbial profiles between athletes with and without HTN. Samples were collected at home by the subject by using the sterile swab collection kit on used toilet tissue. Samples were collected in stool swab collection kits (including preservative solution and kept at room temperature per instructions) within 24 h of the BP measurement, body composition and VO_2_max testing and returned to the laboratory for shipment to uBiome for processing samples. Methodology for isolation and amplification of microbial 16S rRNA by uBiome has been published previously (Almonacid et al., 2017). Utilizing the V4 region of the 16S rRNA gene, microbial sequence data was quality‐filtered and assigned to corresponding samples based on their 12‐bp error‐correcting Golay barcodes using QIIME2 (Bolyen et al., 2019). We received the raw data and barcodes from uBiome to complete our microbial analysis. Actual sequence variants were generated using deblur (Amir et al., 2017) to identify microbial subOTUs, and amplicon sequence variants (ASVs) were analyzed using Quantitative Insight into Microbial Ecology 2 (QIIME2) and R Statistical Software (version 4.2.1).

### 2.6 Statistical analysis

Subject characteristic data are presented as mean ± SD. Variables were analyzed via independent *t*‐tests to assess differences between groups. Statistical significance was set at *p* ≤ 0.05, and statistical analysis was performed with SPSS v.21 (Chicago, IL). Pearson correlations were generated to test the association between continuous variables (physical measures, blood pressure, body composition, and VO_2_max). To visualize pairwise correlations, a correlogram was generated using the R package “cooccur”.

### 2.7 Microbiome analysis

Community evenness (Shannon index), community dominance (Inverse Simpson index), and the observed number of ASVs were employed to measure microbial diversity using the R package “microbiome”. Bray‐Curtis dissimilarities were generated to observe differences in microbial community structure, and a permutational analysis of variance (PERMANOVA) was generated to test the significance in microbial variation between normal versus HTN using the R packages “phyloseq” (McMurdie & Holmes, 2013) and “vegan”. False discovery rate corrected (FDR‐corrected) *p*‐values were generated to test significance (*p* < 0.05) for all permutational analyses. Random forest models (a machine learning classifier) were generated to assess ASVs that differentiated between normal versus HTN using hierarchical decision trees to identify an ASVs importance to the model's accuracy using the R package “randomForest”. Using an out‐of‐bag (OOB) model, each microbial taxa in the training dataset was removed to observe its effect in altering the error rate in classifying participants with normal BP versus HTN. To account for the non‐normal distribution of microbial composition, Spearman rank correlations were calculated for microbial taxa against average systolic BP. Microbial dysbiosis was computed using the R package “dysbiosisR”. Dysbiosis models were generated using the nearest prototype (centroid) classifier measuring the distances between samples labeled as healthy (normal BP) versus disease (HTN) class to assess closeness as described by AlShawaqfeh et al. 2017 (AlShawaqfeh et al., 2017). For the purpose of our study, microbial dysbiosis was calculated using a dissimilarity distance (Euclidean) rather than 10 distinct microbial taxa as referenced by previously (AlShawaqfeh et al., 2017). All visualizations were generated using the R package “ggplot2”.

## 3 RESULTS

### 3.1 Subject characteristics

Thirty participants had a complete data set and high‐quality microbiome data for our analysis (normal BP: *n* = 15; seven males; HTN: *n* = 15; 11 males). Participant characteristics are shown in Table 1. There were men's basketball athletes (11 participants; seven HTN group), track and field athletes (11 participants; three HTN group), women's volleyball athletes (six participants; three HTN group), and men's football athletes (two participants; two HTN group) in this study. Age (years), and BMI (normal, overweight, or obese), fat mass (percentage) and relative VO_2_ max (mL/kg/min) were not statistically different between athletes with normal BP versus HTN. The HTN group did have significantly greater body weight (lbs; *p* = 0.0051), muscle mass (lbs; *p* = 0.0098), and systolic (*p* = 0.0001) and diastolic (*p* = 0.0086) BP. Additionally, a Pearson correlation was conducted among physical measures (weight and height), body composition (percent body fat and muscle mass), blood pressure (avg. systolic and diastolic), and cardiorespiratory fitness (absolute and relative VO_2_max). Expectedly, weight (*R* = 0.34) and percent fat mass (*R* = −0.69) were negatively correlated with relative VO_2_max. However, average systolic blood pressure (*R* = −0.02) and muscle mass (*R* = 0.06) showed no significant correlation to relative VO_2_max among participants (Figure S1 **)**.

### 3.2 Gut microbiome characteristics

**FIGURE 1:**
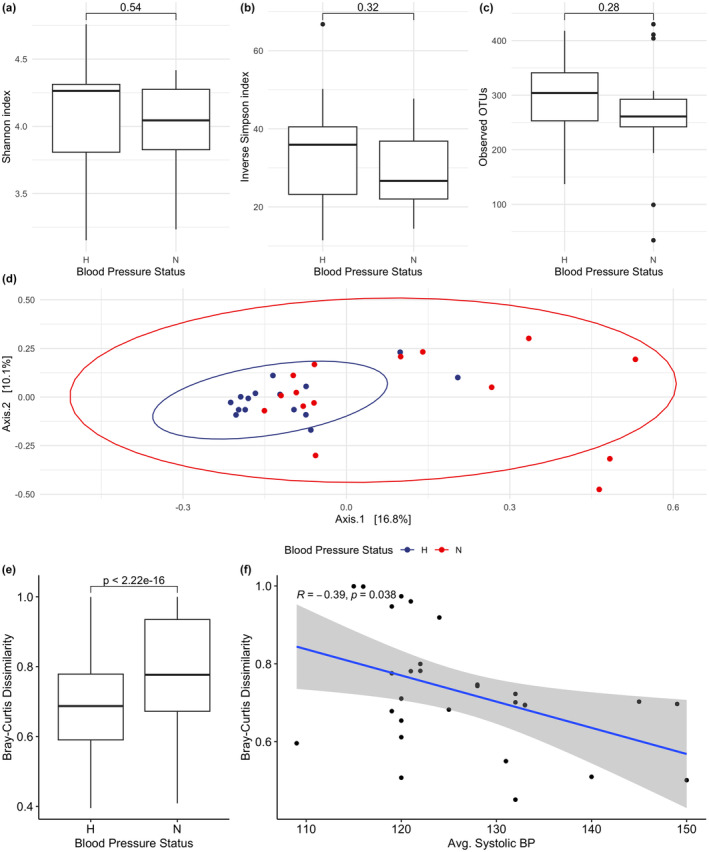
Samples were binned by normal versus high blood pressure and microbial diversity was assessed for differences in community evenness (a), community dominance (b), and the observed number of ASVs (c). Principle coordinate analysis (d) was generated to observe microbial community structure for normal versus high blood pressure. 95% confidence intervals were visualized using ellipses. (e) Bray‐Curtis dissimilarities were binned by blood pressure status and visualized via boxplots. A permutational ANOVA was generated to denote significance of microbial community structure between the two groups. (f) Changes in Bray‐Curtis dissimilarities were plotted against average systolic blood pressure. Spearman rank correlations were applied to test significance, and 95% confidence intervals were shaded in gray.

Following filtering, 1530 ASVs totaling 2,505,358 sequences were identified for our cohort. Among microbial taxa, Firmicutes (60.6%) and Bacteroides (29.4%) comprised approximately 90% of the microbial community among the cohort. Microbial diversity and community structure were analyzed to assess whether BP status was differentially associated with altered microbial variation. For measures of alpha diversity, there were no significant differences (ANOVA, *p* > 0.05) in community evenness (Shannon Index), community dominance (Inverse Simpson index), or the number of the observed OTUs between the two groups (Figure 1a–c). To assess whether microbial community structure (i.e., beta diversity) was different between participants, Bray‐Curtis dissimilarities were analyzed against participant characteristics. Using a permutational analysis of variance (PERMANOVA), no significant difference (*p* > 0.05) was observed when comparing microbial community structure against VO_2_max (both absolute and relative) suggesting microbial variation was not associated with differences in physical fitness. However, a principal coordinate analysis (Figure 1d) showed a significant difference (PERMANOVA, *p* < 0.05, *R* = 0.235; Table S1) when participants were binned into normal BP versus HTN (Figure 1e). To further understand the association of blood pressure on microbial variation, systolic BP was inversely correlated (Spearman, *p* < 0.05, *R* = −0.39) with beta diversity suggesting as BP increased microbial variation was significantly reduced (Figure 1f).

**FIGURE 2:**
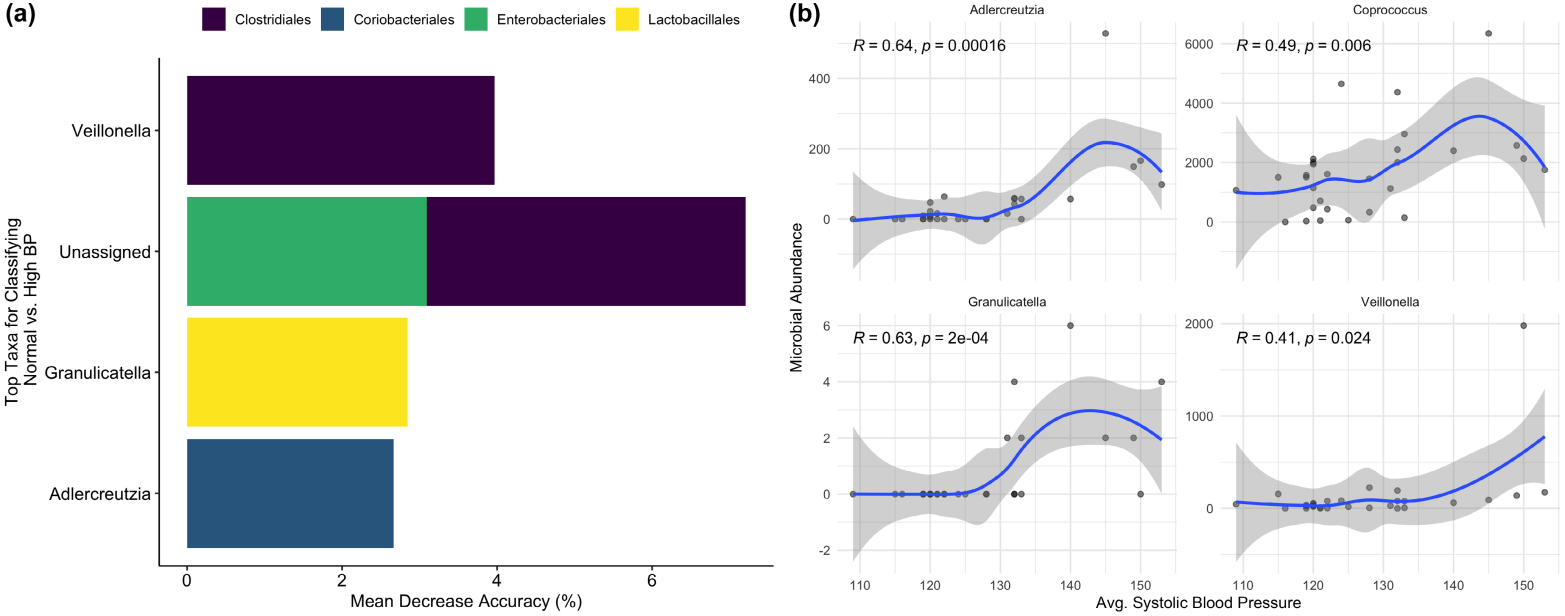
(a) The top 10 ASVs binned at the genus level were reported and visualized from random forest models measuring microbial importance in discriminating between normal versus high blood pressure based on the model's mean decrease in accuracy. (b) Spearman rank correlations were fitted against a generalized regression for ASVs from random forest model to test significance against average systolic blood pressure.

To identify microbial taxa associated with BP status, random forest models were utilized, which are suited for analyzing both sparse and compositional data such as microbial sequence data. Using an out‐of‐bag classification, microbial composition showed improved accuracy when predicting between normal BP versus HTN (AUC = 0.524). Compared to the PERMANOVA, the participants with normal BP versus HTN is better explained by microbial composition via the random forest model. With an overall error rate of 40%, HTN samples were accurately classified at 66.7% while normal BP samples were only accurately classified at 53.3% suggesting microbial community composition is more variable among participants with normal BP. To identify microbial taxa's importance in classifying BP status, error rates were analyzed. Error rates increased when *Adlercreutzia* (2.67%), *Coprococcus* (2.38%), *Granulicatella* (2.84%), and *Veillonella* (3.97%) were excluded from the training model. Additionally, unassigned genera within Clostridiales, Enterobacteriales, and Erysipelotrichales accounted for a 17.5% increase in the error rate collectively (Figure 2a). Spearman rank correlations were employed to further investigate microbial taxa important in discriminating between participants with normal BP versus HTN. Assuming a non‐normal distribution of microbial composition, Spearman correlations showed a significant (*p* < 0.05) direct associations between systolic BP and abundances for *Adlercreutzia* (*R* = 0.64), *Coprococcus* (*R* = 0.49), *Granulicatella* (*R* = 0.63), and *Veillonella* (*R* = 0.41) (Figure 2b). These findings suggest the random forest model serves as a better predictor of variances in gut microbiota between participants with normal versus high BP when compared to the PERMANOVA (*R* ^2^ = 5.5%) suggesting the machine learning classifier is a better model for identifying keystone microbial taxa.

**FIGURE 3:**
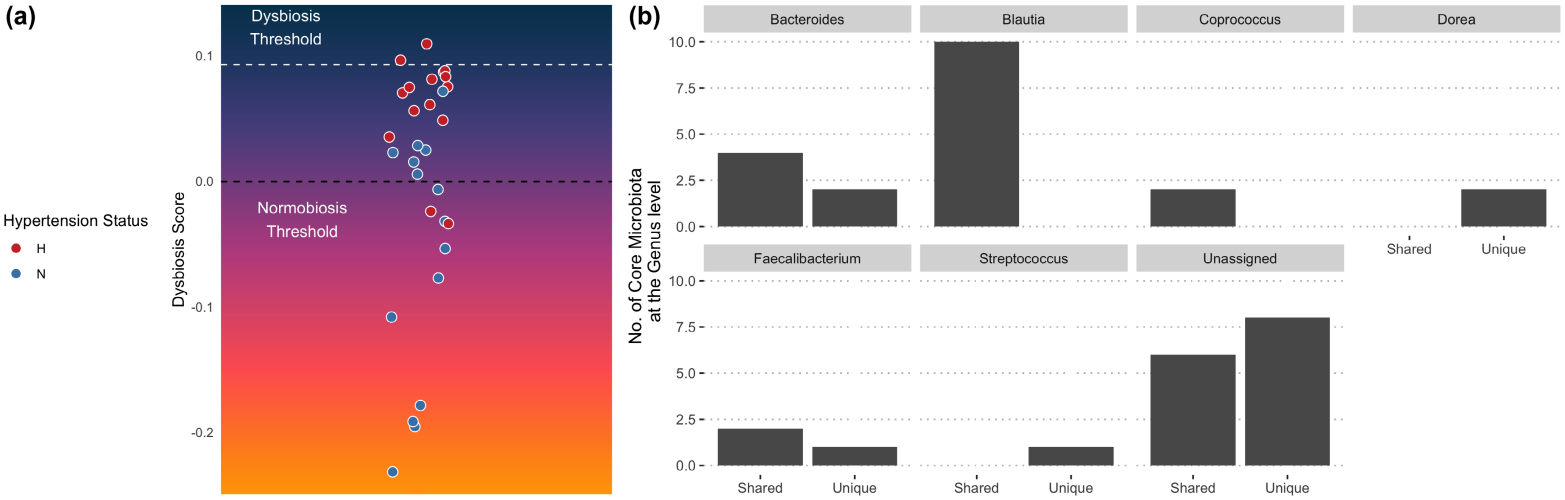
(a) A gradient plot was generated to visualize microbial dysbiosis among individuals. Quantiles were visualized to observe dysbiotic versus normobiosis. Samples were colored by blood pressure status. (b) 38 ASVs were identified as core microbial taxa and binned at the genus level and visualized to show whether the ASVs were unique to HTN or shared between the two BP statuses.

In addition to analyzing differences in microbial taxa between normal BP vs. HTN, microbial composition was used to compute taxa co‐occurrences associated with participant groups, which we termed as a microbial dysbiosis index for this study. Dysbiosis was computed using Euclidean distances models to measure closeness among participants with normal BP vs. HTN. Thirteen out of 15 (86.7%) HTN samples were classified as dysbiotic and the remaining samples were classified as healthy (Figure 3a). Overall, the model was highly accurate (AUC = 0.911) discriminating between normal BP versus HTN based on dissimilarities in microbial structure (Figure S3). To further assess microbial taxa associated with HTN, a Venn diagram (Figure S2) was generated to observe the number of microbiota unique to normal BP versus HTN, as well as the number of shared microbes between the 2 BP statuses. Observing microbial taxa with a relative abundance of 1% and present in at least 75% of the samples, 38 ASVs were either unique to HTN or shared between normal BP and HTN. However, no ASVs were unique to normal BP. When ASVs were classified at the genus level, the genera *Bacteroides* (*n* = 2), *Dorea* (*n* = 2), *Faecalibacterium* (*n* = 1), *Streptococcus* (*n* = 1), and unclassified genera (*n* = 8) were among ASVs unique to HTN. Separately, the genera *Bacteroides* (*n* = 4), *Blautia* (*n* = 10), *Coproccus* (*n* = 2), *Faecalibacterium* (*n* = 2), and unclassified genera (*n* = 6) were shared between normal BP and HTN (Figure 3b).

## 4 DISCUSSION

To our knowledge, this is the first study to assess and report gut microbiome characteristics associated with BP status in AA collegiate athletes. In this cohort of athletes, our significant findings are that microbial alpha diversity was not different, but community structure (beta diversity) was different stratified by BP status. We did not observe any differences in relative VO_2_max or VO_2_max associations with microbial characteristics between the groups. Further, microbiome composition assessment revealed that the microbial genera *Veillonella*, *Adlercreutiza*, *Coprococcus*, and *Granulicatella* were directly associated (i.e., more abundant) with greater systolic BP. Combined, the dysbiosis index and the identification of ASVs largely associated with blood pressure status may serve as important biomarkers central in structuring microbial interactions associated with BP status. The results of this study will aid in directing a deeper dive into the function of these microbes, their metabolism, and identify potential interventional strategies (e.g., nutrition) to improve the dysbiosis profile and lessen CVD risk.

In a recent and timely review, O'Brien et al. (O'Brien et al., 2022) discuss the interdependent relationship between exercise and gut microbial characteristics that may benefit athletic performance and that impact health differentially during times of moderate versus intense exercise participation. However, there is underwhelming attention on gut microbial characteristics associated with disease risk in this population.

### 4.1 Subject and gut microbiome characteristics

In AA athletic populations, the association between BP and fitness has not been well‐defined even with the increased risk of HTN. Even less understood is the interaction between the gut microbiome on BP status, which was the focus of this study. Studies have reported the relationship of body weight and muscle mass in multiple groups with the most recent human study showing the positive association of lean body mass with BP in young adults (Vaziri et al., 2015). However, in this cohort of both aerobic and anaerobic trained athletes, we did not observe any significant association with body weight and muscle mass with any microbial communities.

Concerning gut microbial characteristics, we did not observe any significant differences in alpha diversity indices or SCFA‐producing microbial abundances with BP status, as we hypothesized that we would. This result implies that the gut microbiome, in highly physically fit collegiate athletes, does not follow the same pattern of gut dysbiosis associated with HTN in sedentary individuals and is likely explained by chronic exercise's ability to maintain a highly diverse microbial profile (Mailing et al., 2019). Further, increased BP was associated with beta diversity (community structure) and imply that there is a restructuring of the microbial community by reducing the variation among athletes with HTN when compared to those exhibiting normal BP.

### 4.2 Microbial taxa and blood pressure

Our direct and exploratory analyses revealed that the microbial genera *Veillonella*, *Adlercreutiza*, *Coprococcus*, and *Granulicatella* were directly associated (i.e., more abundant) with greater systolic BP. *Veillonella* is a bacterial group typically found in the human gut (Lee et al., 2021) that has been shown to functionally degrade lactate, a byproduct of anerobic metabolism (Cairns, 2006), in elite athletes (Scheiman et al., 2019). *Veillonella* has also been associated with the oral microbiome, as a nitrate reducing microbe, and was suggested to participate in altering circulating nitrite bioavailability for BP control (Pignatelli et al., 2020; Vanhatalo et al., 2018). One study reported a direct correlation with oral *Veillonella* abundance and HTN status (Sohail et al., 2021). In the most recent study of individuals of African origin, Fei et al. (Fei et al., 2019) reported that oral *Veillonella* was associated with increased cardiometabolic risk in their cohort. In the stool, it was reported to be an opportunistic pathogen that was associated with dysbiosis and elevated BP in women with pre‐eclampsia (Chen et al., 2020) but physical activity status of the individuals in that study was not reported.

Given the emerging evidence surrounding this bacterium and its relationship with BP, our study is the first to report stool *Veillonella* abundances direct association with high systolic BP in athletes (Figure 2). During training and competition, athletes can chronically produce significant amounts of lactate that are usually cleared by tissues during recovery and completely oxidized or reconverted to glucose in the liver via the Cori cycle (Yang et al., 2020). Interestingly, the gut microbiome may adapt to participate in the clearance of lactate, and this could explain the greater abundance of *Veillonella* given its ability to process lactate (Grosicki et al., 2019; Scheiman et al., 2019). Thus, *Veillonella*'s association with BP could be more related to trained status (e.g., anaerobic vs. aerobic) than BP status itself. To tease out this relationship, future studies are needed to characterize *Veillonella* abundance separately in aerobic and anaerobically trained athletes.

#### 4.2.1 Coprococcus

*Coprococcus* are anaerobic species whose abundance in the gut microbiome has been previously associated with BP status. Specific taxa of this genus were discovered to be carbohydrate fermenters and SCFA producers (i.e., butyrate, propionate, and acetate) (Lillian & Holdeman, 1974) which can be viewed as a beneficial occurrence and could explain this genus' abundance being positively associated with normal BP (Dan et al., 2019). This genus could be an exercise adaptable microbial group to expand SCFA production, which could be common in exercise trained groups (Mailing et al., 2019). However, greater *Coprococcus* abundance was associated with elevated BP in this cohort of athletes. In our critical assessment of this result, it is possible that the increased abundance is (1) a normal response to chronic exercise training to increase/sustain SCFA production, (2) a compensatory increase in SCFA producing taxa of this genus related to elevated BP in effort to resist further increases in BP, or (3) an expansion of species that are not SCFA producers and may be more “opportunistic” and therefore promote cardiovascular dysfunction(s). The best approach to investigate this further is to perform whole genome sequencing of gut microbial samples and identify specific taxa that are associated with BP status. Unfortunately, we are not able to perform that analysis in this study but have plans to in future investigations.

#### 4.2.2 Adlercreutiza

Previous studies have reported that *Adlercreutiza* was correlated with greater BMI and inflammation in obese individuals (Dekker Nitert et al., 2020). Microbial taxa of this genus have also been associated with greater dietary flavanol and flavanone consumption (Ivey et al., 2019). In our cohort, *Adlercreutiza* abundance was shown to be higher in the HTN group. As this genus' abundances have been correlated with BMI, inflammation, and dietary habits, our results are in line with the association with BMI as our HTN subjects BMI was greater. Three‐day dietary recall nutrient intake and association with BP has been recently reported by our group in most of the athletes in this cohort (Purdom et al., 2023). However, we were not able to comprehensively assess microbiome characteristics and nutrient intake in all participants. In future studies, dietary assessment will be important to determine if there are micro‐ or macro‐nutrient differences that are associated with the greater abundance of *Adlercreutiza* in athletes with HTN.

*Granulicatella* species are a part of the human gut microbiome (Christensen & Facklam, 2001). In the context of cardiovascular disorders, microbial taxa of *Granulicatella* genus have been associated with some incidences of infective endocarditis (Estevez et al., 2022). From the current literature, we can only deduce that some species are opportunistic pathogens, and their presence could be a factor in microbial dysbiosis associated with HTN in this cohort. Additional studies are needed to confirm if this is a commonly abundant species in a dysbiotic gut or if their abundance is regional/environmental.

#### 4.2.3 Blautia and Dorea

Microbes of the genus *Blautia* have been previously associated with elevated BP in cross‐sectional animal and human studies (Yan et al., 2022). Blautia microbes metabolize carbohydrates with main end‐products being acetic acid, succinic acid, lactic acid, and other SCFAs (Freitas et al., 2022; Liu et al., 2021). In Figure 3b, our analyses show that 10 ASVs within the *Blautia* genus were shared between the microbiome of athletes with normal and high blood pressure. As exercise increases the abundance of SCFA producing bacteria (Allen et al., 2018; Mailing et al., 2019), *Blautia* may be an exercise‐responsive genus in groups that perform chronic exercise training as their increased abundance may be related to optimization of carbohydrate metabolism. In a short (3‐week) high‐intensity exercise intervention by Rettedal et al. (Rettedal et al., 2020) that included lean and overweight sedentary individuals, researchers reported that *Blautia* and *Dorea* were significantly associated with insulin sensitivity indices (plasma insulin and homeostatic model assessment of insulin resistance (HOMA‐IR)) in their overweight subjects only. We also report in Figure 3b that two *Dorea* ASVs were unique to the HTN group and bacteria in this genus have been shown to be responsive to (e.g., increased) exercise and dietary intervention (low carbohydrate/high fat) (Clauss et al., 2021). Interestingly, *Coprococcus* (discussed above) was also positively associated with plasma insulin and HOMA‐IR in the previously mentioned study (Rettedal et al., 2020). As insulin resistance and HTN are often associated with one another, there may be common microbes shared in between metabolic and HTN dysbiosis profiles. However, our analysis of microbial dysbiosis is a correlative observation in microbial occurrences associated with participants classified as HTN rather than an actual biomarker of disease. An increase in sample size and further analysis is needed to demonstrate causation. Last, we were not able to collect blood in our study but future studies will require comprehensive assessment of blood biomarkers (i.e., glucose, insulin sensitivity, and lipids) and gut microbial composition to compare pathophenotypes associated with metabolic and vascular dysfunction in this population.

### 4.3 Limitations

We acknowledge that differences in sport specific training that necessitate different training regimens and intensities (anaerobic vs. aerobic athletes) may have a role in gut microbiome characteristics. Time to last exercise bout (Grosicki et al., 2023) and time of the day when fecal samples were self‐collected could impact variability between participants, but all samples were collected within 24 h of the testing day. Further, the sample size may be suboptimal, but we were able to identify statistically significant differences in microbial abundances after correction for multiple comparisons. Associations between nutrient intake and microbiome characteristics would provide deeper insight into the function metabolic profiles of the microbiome associated with BP status. Future studies will include comprehensive dietary recall assessment, blood biomarkers, sleep quality data, social determinants of health (e.g., psychological/perceived stress load, perceived racism, housing, access to food, etc.), and whole genome shotgun sequencing of gut microbial samples to identify specific taxa and metabolomic profiles associated with BP status.

## 5 CONCLUSION

As HTN is common in some athletic populations, studies are needed to plan effective interventions that improve BP and preserve performance. Pharmacological interventions for BP in athletes may negatively impact performance due to their mechanisms of action, such as altering fluid balance, smooth muscle contractile properties, and cardiovascular electrical activity (e.g., diuretics, calcium channel, and beta blockers, respectively). Therefore, more research is needed to improve CVD risk by defining associations between dietary habits, microbiome characteristics, and BP status. With this, interventions can then be introduced for microbiome targeted strategies to alleviate this burden and provide a path to discovery of exercise adaptable species associated with cardiovascular health.

## AUTHOR CONTRIBUTIONS

TH and JH‐M were involved in data analysis, writing, editing, and approval. IC was involved in data analysis, editing, and approval. TE and MB were involved in writing, editing, and approval. TP and HC were involved in data analysis, editing, and approval. MDC was involved in conception and design, data analysis, writing, editing, and approval.

## FUNDING INFORMATION

Awarded to Marc Cook. American Heart Association (Career Development Award: 18CDA34110444).

## CONFLICT OF INTEREST STATEMENT

The authors have no conflict of interests to disclose.

## ETHICS STATEMENT

This study was approved by the North Carolina A&T State University Institutional Review Board (IRB: 17‐148). All participants completed written informed consent before data collection.

## Supporting information


Figure S1.

Figure S2.

Figure S3.

Table S1.

## Data Availability

Data are publicly available here: (to add if accepted). Private link has been shared.
