# Supplementary material for: Gut microbiota are differentially correlated with blood pressure status in African American collegiate athletes: A pilot study
Source: Physiol Rep. 2024 Mar 21;12(6):e15982. doi: 10.14814/phy2.15982 (PMC10957718; doi:10.14814/phy2.15982)
Supplement: Supplementary file 1 — Figure S1. [file PHY2-12-e15982-s004.zip › Supplemental Figure 1.docx]

**Supplemental Figure 1**. A correlogram was generated for participant characteristics associated with blood pressure (average diastolic and systolic), body composition (percent fat mass and muscle mass), cardiorespiratory fitness (absolute and relative VO2max), and physical measures (weight and height). Pearson correlations were generated for pairs and colored by the strength of their correlation (red = -1, white = 0, and blue = 1).
