# Supplementary figures and images for: Gut microbiota are differentially correlated with blood pressure status in African American collegiate athletes: A pilot study
Source: Physiol Rep. 2024 Mar 21;12(6):e15982. doi: 10.14814/phy2.15982 (PMC10957718; doi:10.14814/phy2.15982)

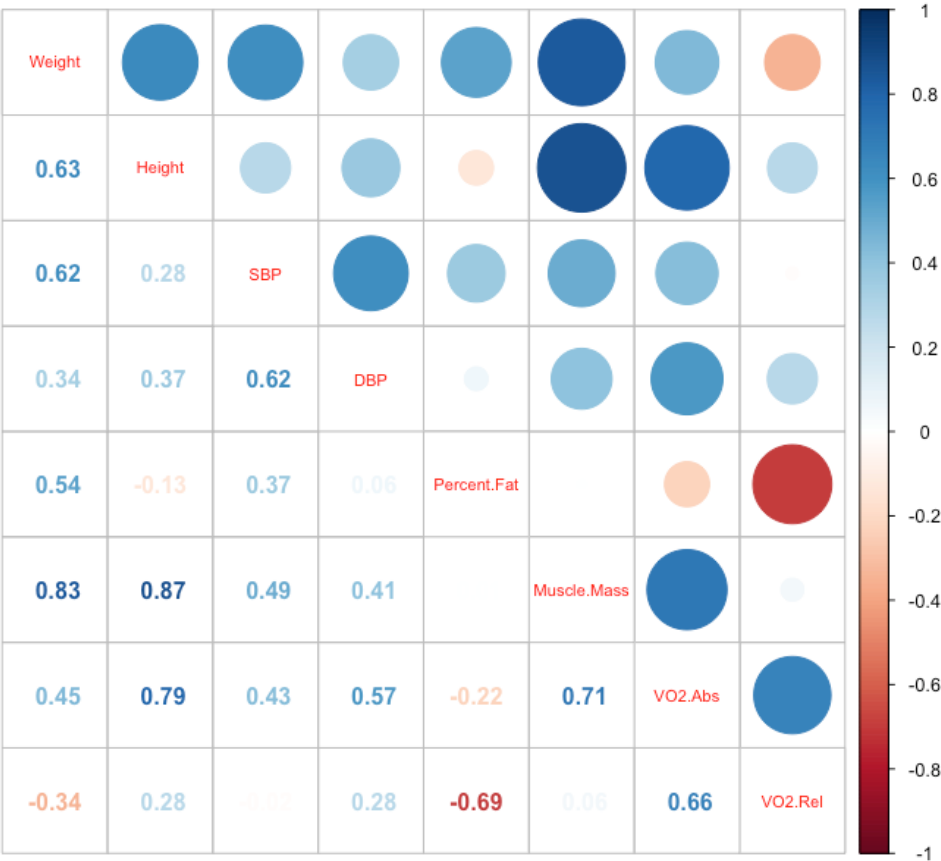

Supplement: Supplementary file 1 — Figure S1. [file PHY2-12-e15982-s004.zip › phy215982-sup-0001-FigureS1 .pdf]

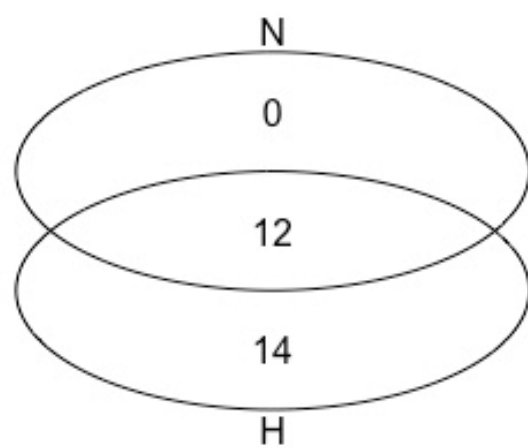

Supplement: Supplementary file 2 — Figure S2. [file PHY2-12-e15982-s002.zip › phy215982-sup-0002-FigureS2 .pdf]

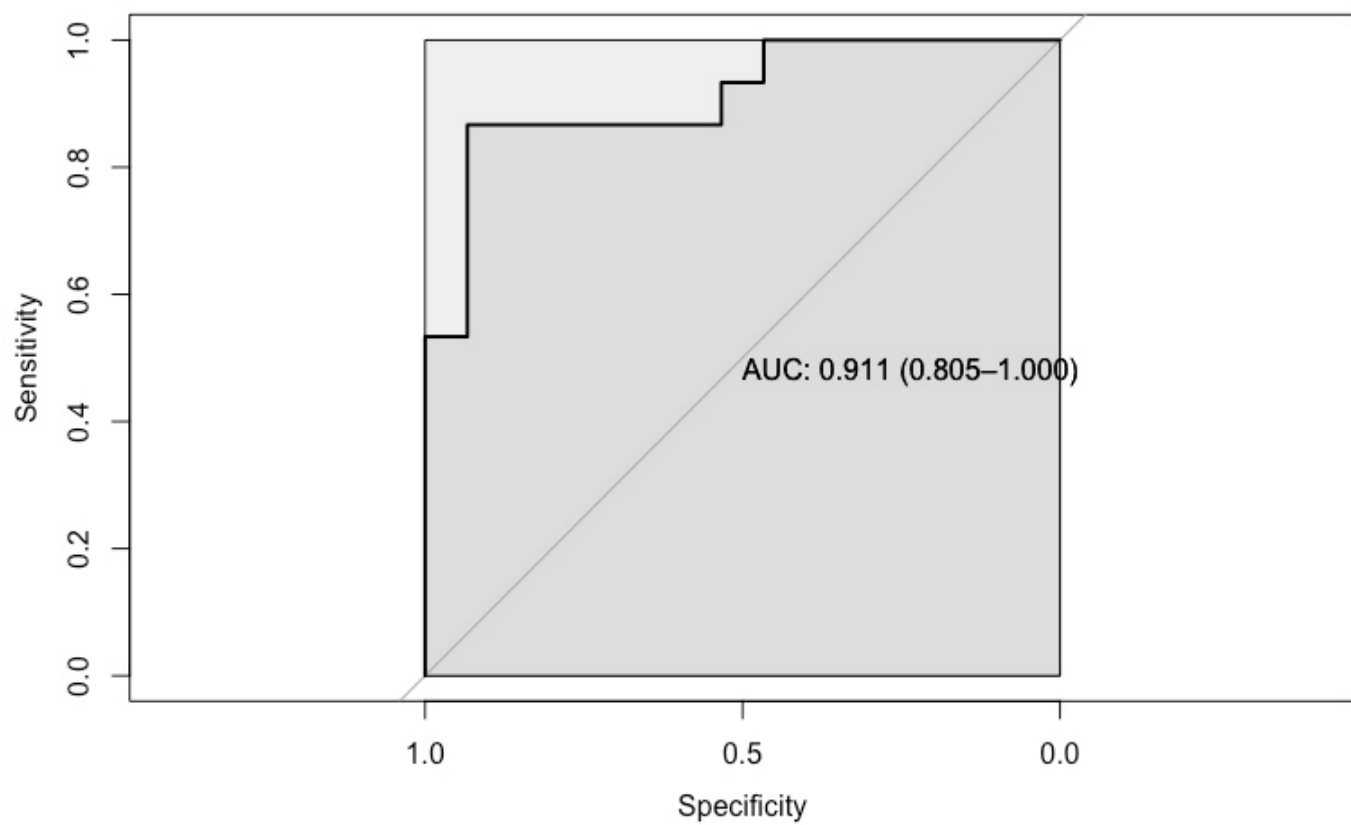

Supplement: Supplementary file 3 — Figure S3. [file PHY2-12-e15982-s001.zip › phy215982-sup-0003-FigureS3 .pdf]
