# Supplementary material for: Gut microbiota are differentially correlated with blood pressure status in African American collegiate athletes: A pilot study
Source: Physiol Rep. 2024 Mar 21;12(6):e15982. doi: 10.14814/phy2.15982 (PMC10957718; doi:10.14814/phy2.15982)
Supplement: Supplementary file 2 — Figure S2. [file PHY2-12-e15982-s002.zip › Supplemental Figure 2.docx]

**Supplemental Figure 2**. A Venn diagram was generated to show the number of unique taxa in participants with normal BP vs. HTN, as well as those shared between the two. Only microbial taxa whose relative abundance was at least 1% and prevalent 75% were included.
