# Supplementary material for: Gut microbiota are differentially correlated with blood pressure status in African American collegiate athletes: A pilot study
Source: Physiol Rep. 2024 Mar 21;12(6):e15982. doi: 10.14814/phy2.15982 (PMC10957718; doi:10.14814/phy2.15982)
Supplement: Supplementary file 3 — Figure S3. [file PHY2-12-e15982-s001.zip › Supplemental Figure 3.docx]

**Supplemental Figure 3**. Accuracy for predicting normal BP vs. HTN was assessed using an area under the curve (AUC) values, which measures the model’s sensitivity and specificity. 95% confidence intervals shaded in gray were generated to assess deviations in these value when predicting microbial dysbiosis based on blood pressure status.
