# Supplementary material for: Gut microbiota are differentially correlated with blood pressure status in African American collegiate athletes: A pilot study
Source: Physiol Rep. 2024 Mar 21;12(6):e15982. doi: 10.14814/phy2.15982 (PMC10957718; doi:10.14814/phy2.15982)
Supplement: Supplementary file 4 — Table S1. [file PHY2-12-e15982-s003.zip › phy215982-sup-0004-TableS1.docx]

|  | **DF** | **Sum of Squares** | **R2** | **F** | **Pr(>F)** |
| --- | --- | --- | --- | --- | --- |
| **Hypertension** | 1 | 0.4535 | 0.05512 | 1.6334 | 0.01 ** |
| **Residual** | 28 | 7.774 | 0.94488 |  |  |
| **Total** | 29 | 8.2275 | 1 |  |  |
