# Supplementary material for: Gut microbiota are differentially correlated with blood pressure status in African American collegiate athletes: A pilot study
Source: Physiol Rep. 2024 Mar 21;12(6):e15982. doi: 10.14814/phy2.15982 (PMC10957718; doi:10.14814/phy2.15982)
Supplement: Supplementary file 4 — Table S1. [file PHY2-12-e15982-s003.zip › Supplemental Table 1.docx]

**Supplemental Table 1**. Describes a summary of the permutational analysis of variance when associating blood pressure status via variances in microbial community structure. An FDR-corrected p-value < 0.05 was used to denote significance.
